# A comparison of the marginal adaptation of composite overlays fabricated with silicone and an intraoral scanner

**DOI:** 10.4317/jced.58140

**Published:** 2021-05-01

**Authors:** Carla García-Cuesta, Vicente Faus-Llácer, Álvaro Zubizarreta-Macho, René Botello-Torres, Vicente Faus-Matoses

**Affiliations:** 1 PHD student. Department of Endodontics, Faculty of Medicine and Dentistry, University of Valencia, Spain; 2 Director of the master’s Program in Endodontics. Department of Endodontics, Faculty of Medicine and Dentistry, University of Valencia, Spain; 3 Department of Endodontics, Faculty of Health Sciences, Alfonso X El Sabio University, Madrid, Spain; 4 Professor of the master’s Program in Endodontics. Department of Endodontics, Faculty of Medicine and Dentistry, University of Valencia, Spain; 5 Co-director of the master’s Program in Endodontics. Department of Endodontics, Faculty of Medicine and Dentistry, University of Valencia, Spain

## Abstract

**Background:**

Intraoral scanners have been developing during last years. The aim of this study was to know if digital impressions achieve a marginal adaptation in overlays as well as conventional impressions with silicone.

**Material and Methods:**

Sixty-two extracted molars were selected. The samples were prepared for MOD overlays. The teeth were divided into two groups (n=31). Group 1: impressions were made with silicone (Express™ Impression, 3M ESPE) and overlays were manufactured with Sinfony composite (3M ESPE) by the laboratory technician. Group 2: impressions were taken with the scanner True Definition (TD, 3M ESPE) and under Lava Ultimate CAD/CAM Restorative composite (3M ESPE) were produced the restorations. Under 32x magnification images of vestibular, lingual, mesial and distal were capture in all the samples. Then the fit of the restorations was evaluated before and after cementation. Data were analysed statistically applying ANOVA and Bonferroni test.

**Results:**

The marginal gap was better in TD group before (169,76 ± 54,15 µm) and after (145,16 ± 57,89 µm) cementation than in the silicone group (190,89 ± 58,18 µm) (187,47 ± 81,29 µm). The lowest marginal gap was in oclusal surface and the higher value was in the proximal margin for all the groups.

**Conclusions:**

Digital impressions regarding marginal adaptation achieve better results than conventional impressions.

** Key words:**Composite onlays, overlays, restorative, CAD/CAM, intraoral scanner, silicone impressions, marginal adaptation.

## Introduction

Direct restorations are use in large cavities giving good results. Despite this, over the years there were introduce the indirect restorations, providing a better oclusal anatomy, contact points and more fracture resistance (1-3). The way to manufacture overlays has been improved increasing the characteristics of those restorations. The CAD/CAM systems, digital design and manufacturing accelerated the working flow, the patient comfort and obtain good results (4). Presently there are more than 20 materials for using in milling machine, presented in blocks, with different size, shape, translucency and colour (4).

Making a good intraoral impression is crucial for the final restoration and is one of the most difficult step in dental practice (5). The accuracy of the impressions depends on the material used, the kind of the tray and the technique. Every step in the workflow could lead out to error (6). A good impression material has to achieve some properties as a reproducibility, elastic recovery, dimensional stability, flexibility, simple handling, hydrophilic, conforTable for the patient and economic (7). Until now there are some impression materials that have been used. Nowadays intraoral scanners can be considered as a new method for impressions. At this time the scanners more used are the CEREC System (Sirona Dental Systems), Lava C.O.S System (3M ESPE), iTero System (Cadent/Straumann), E4D System (D4D Technologies) y Trios System (3D Shape) (8,9).

The scanner True Definition (TD) uses “active wavefront sampling” creating the concept “3D in Motion” which led to capture 20 images per second creating a three-dimensional model. This system needs to cover the surface with a light coating of titanium-oxide powder prior to scanning (10).

The material of the restoration, the impression technique and the cementation influence in marginal adaptation of indirect restorations. Holmes *et al.* in 1989 defined the marginal gap as the measurement between the axial wall of the prepared tooth and the internal surface of the restoration (11). In several articles the size of the marginal gap has to be among 100 or 150 µm. However another studies consider acceptable a gap of 110 or 200 µm. Nevertheless there are not an exactly value to determine the size of the marginal gap (12-14).

The marginal gap as well as the internal gap could influence in the longevity of the restorations, in their wear, filtration or cement erosion. When the space between the tooth and restoration increases, the resistance to fracture decreases. So an elevate misfit allows the exposure of the cement what can bring a dissolution of it (12,13).

It is relevant to know about the use of intraoral scanners and if they provide some advantages against conventional impressions. The aim of this study was to compare the marginal fit of indirect restorations obtained by a conventional and digital method. Also was to contrast the differences between groups before and after cementation and between different study locations. The null hypothesis was that no difference would be found in marginal fit of restorations fabricated with conventional and digital method.

## Material and Methods

- Specimen selection

After the approval of the Ethics Committee with Nº H1523549290042, sixty-two extracted molar were selected. The study was done in the Master en Odontología Restauradora y Endodoncia, Department of Stomatology, Faculty of Medicine and Dentistry, Valencia, Spain. It was done *in vitro* under ideal conditions. All samples were mounted in plaster up to 2 mm apical to the cement-enamel junction.

- Specimen preparation

**Figure 1:**
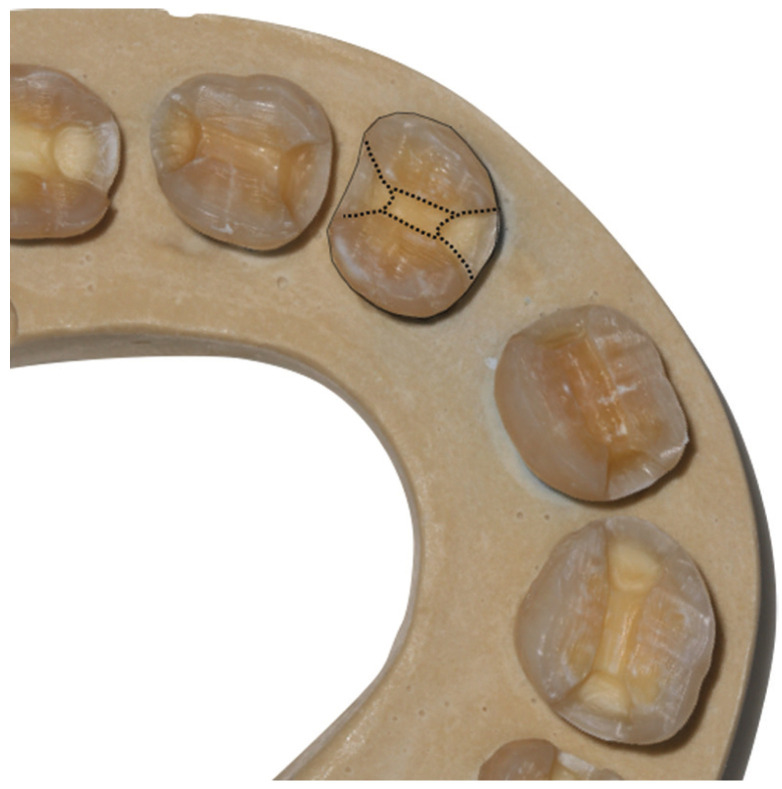
MOD preparation for overlays.

MOD cavities for overlays were prepared in all the samples. A tapered diamond bur (ISO 648-845-314-010K, Komet Dental, Lemgo, Germany) and a rugby diamond bur (ISO 6379 023, Komet Dental, Lemgo, Germany) were used. The oclusal zone was reduced around 1 mm following the dental anatomy. Two marginal boxes were done, one in mesial and the other in distal 2 mm depth. The angles were rounded and the walls were divergent (15). Joining it was made a central box 1 mm depth, obtaining MOD cavities. All of the samples were divided into groups of ten teeth imitating a dental arch and fixing them in plaster following the line made before. In Figure 1 is represented the preparation.

After prepare the entire specimen they were divided into two groups. Group 1: n=31. The impressions were taken with silicone (Express™ VPS Impression Material, 3M ESPE, Seefeld, Germany), a mixture of heavy, medium and light silicone following the two-step technique with plastic trays. The impressions were sent to the lab where the technician manufactured the overlays with laboratory composite, Sinfony. (3M ESPE, Seefeld, Germany). Group 2: n=31 the impressions were taken using the intraoral scanner True Definition (TD, 3M ESPE, St.Paul, MN). Before scanning with the TD teeth need to be dusted with titanium-oxide powder to avoid the refraction of light. The data were transfer to the dental laboratory in STL format where they mill the final restoration using prefabricated blocks of Lava Ultimate Restorative CAD/CAM (3M ESPE, Seefeld, Germany) using the Sirona MCXL milling system.

- Marginal analysis

Once the overlays were prepared the marginal gap was measure before and after cementation. Before cementation the overlays were left in the samples using a light layer of adhesive just to maintain the restoration in the correct position. In all the specimens were painted 16 points in each zone: 5 in vestibular, 5 in lingual, 4 in the proximal zone (mesial and distal) and 2 in gingival zone. By this way the same points were measured before and after cementation.

**Figure 2:**
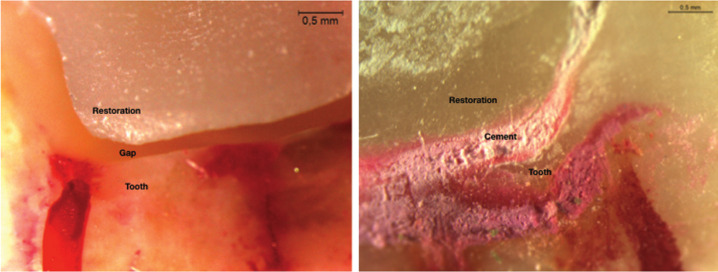
View of the restoration under microscope before and after cementation.

Under a microscope at 32x of magnification MZ APO (Leica Microsystems Inc, Buffalo Grove, IL) images of oclusal, mesial, distal and lingual were capture and transfer to the computer, (Fig. 2). This technique was a non invasive method (16).

Firstly was evaluated the passive adjustment. 16 images per tooth were obtained. Following the gap was measured in microns (µm) the distance between the dental wall and the internal surface of the restoration, using the Power Point (Microsoft office, 2010).

- Cementation of overlays

The internal surfaces of overlays were sandblasted with 50 µm aluminum oxide particles. Later 37% phosphoric acid gel Scotchbond Universal Etchant (3M ESPE, Seefeld, Germany) was left during 15 seconds. It was rinsed and dried and a layer of silane (Relyx ceramic primer, 3M ESPE, Seefeld, Germany) was brushed in the inner surface of the restorations during 1 minute. After that was coat the adhesive Scotchbond Universal Adhesive (3M ESPE, Seefeld, Germany). On the other hand in the teeth was applied firstly the phosphoric acid gel and after the adhesive. Finally all the restorations were cemented using RelyX Ultimate Adhesive Resin Cement (3M ESPE, Seefeld, Germany). After cementation all the specimen were examined under the microscope capturing images of all the surfaces, (Fig. 2).

- Statistical analysis

The data were analysed statistically using the software SPSS 22 (IBM, Inc, Chicago, Illinois, USA). Differences between marginal adaptation before and after cementation and between different locations were evaluated using repeated measure analysis of variance (ANOVA) and Bonferroni test. The level of significance was set at *p* < 0.05.

## Results

The media and standard deviation about marginal adaptation before cementation are represent in Table 1. Before cementation TD group obtained less marginal gap (169,76 ± 54,15 µm) than silicone group (190,89 ± 58,18 µm).

**Table 1:**
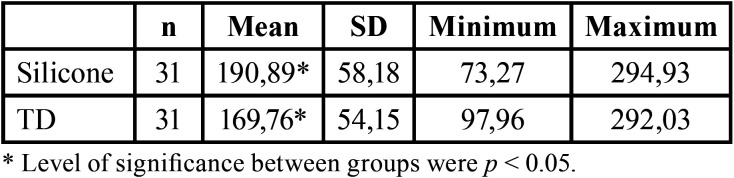
Marginal gap obtained before cementation.

**Figure 3:**
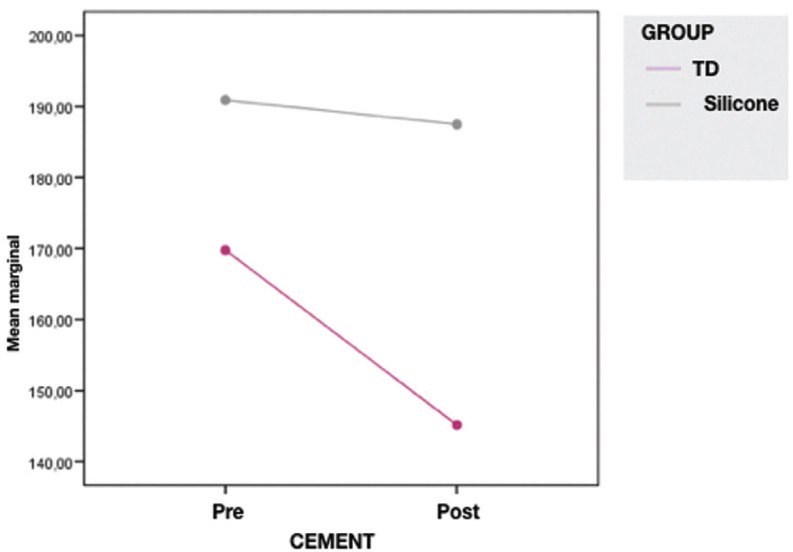
Values of marginal gap before and after cementation.

The media and standard deviation about marginal adaptation after cementation are represented in Table 2. After cementation, the scanner (145,16 ± 57,89 µm) achieved better results regarding marginal adaptation than silicone (187,47 ± 81,29 µm), (Fig. 3).

**Table 2:**
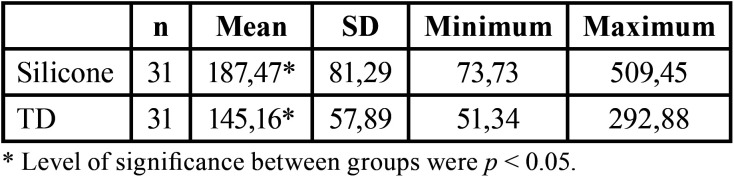
Marginal gap obtained after cementation.

**Figure 4:**
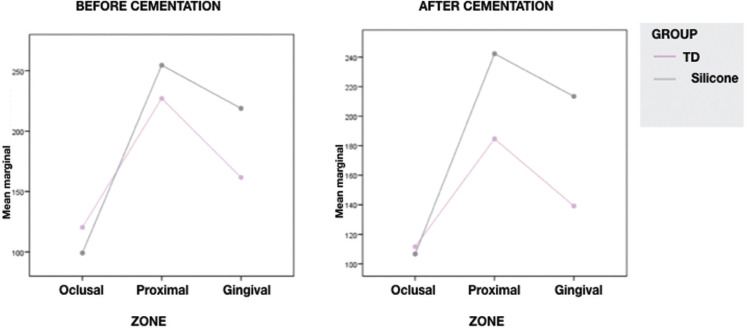
Marginal gap in different zones before and after cemention.

Considering the marginal adaptation among different zones the results showed more adjustment in olcusal one, being similar in both groups without sadistically differences between groups. In proximal and gingival the differences are significant, TD achieves better adaptation than silicone, (Fig. 4).

## Discussion

The results of this study reject the null hypothesis that no differences would be found in marginal adaptation of overlays fabricated with conventional and digital method. Analysis of the results suggested that the scanner TD achieved a better fit before and after cementation. These results were in concordance with other studies where the digital impressions had reached better results in marginal fit comparing it with conventional impressions. Oz *et al.* showed differences in marginal fit of overlays fabricated with a conventional system 85,34 µm (silicone) and with a digital scanner 33,54 µm; 34.23 µm; 33.77 µm (Omnicam). These values expose a misfit in the conventional group higher than in the other one (17). Syrek *et al.* suggested that impressions for crowns get with the scanner Lava C.O.S were able a better marginal adaptation, 49 µm, comparing them with a conventional system, 71 µm. These could be because with the conventional method is need a model to prepare the restorations. For these reason all the steps in the workflow could interfere in the accuracy of the final restoration (18). Ng *et al.* studied the fit of 30 crowns obtained with a scanner and with silicone. The group of the scanner got better marginal adaptation (48 ± 25 µm) than that obtained with silicone (74 ± 47 µm) (19).

On the other hand, some studies did not find any statistically differences between silicone and scanner. Rippe *et al.* analysed the marginal internal and external fit in overlays created with intraoral scanner Lava C.O.S and scanner Bluecam comparing them with silicone. Concluded that all the systems achieved accepTable results without any differences between each other (15).

Cementation could have an influence in the values of adaptation depending on the viscosity of the cement (3). Before cementation all the samples had a space for the cement and for the support of the polymerization shrinkage. Nawaflesh reported a significantly higher marginal gap after cementation than before cementation. This could be affected by the cementation technique such as the uncontrolled finger pressure or over filling the restoration with cement that can cause an uneven flow of cement. Also the type of cement could influence in the fit of the restoration (20). Guess *et al.* demonstrated higher marginal gap after cementation in ceramic overlays. Showed values of 35-50 µm before cementation and 49-63 µm after cementation (21). In this study in the group TD the adaptation was better after cementation (145,16 ± 57,89 µm) and in the silicone group there were not any differences before and after cementation (187,47 ± 81,29 micras). These could be because before cementation the overlays were just let into the tooth without any pressure and after cementation the pressure was done to fix the restoration over the tooth.

The preparation design is known that can influence in the fit of the restoration giving different values depending on the zone (22). In a systematic review conducted by Goujat *et al.* concluded that gingival and axial surfaces of the restoration had greater discrepancy regarding marginal gap (23). In all the groups of the present study the higher misfit was obtained in proximal zone against the oclusal zone, which achieved the best values in marginal fit. In the oclusal zone there were not statistical differences between groups. Similar results were found in the work of Zarrati *et al.* where a greater marginal gap appeared in the gingival edge (70,20 µm) than in oclusal (45,54 µm) or proximal (40,54 µm) (16). Lima *et al.* also reported better results in the oclusal zone (22). More studies are needed to know the accuracy and the precision of scanners in concept of marginal adaptation, to know if they have some advantages against conventional systems.

## Conclusions

Within the limitations of this study the conclusions were:

1. The intraoral scanner achieved better marginal adaptation.

2. In TD, the marginal gap decreases with the cementation (*p*=0,022). In the silicone group were not any differences between cementation and not cementation (*p*=0,745).

3. In all of the groups the marginal gap values were lower in the olcusal zone, followed by gingival. The higher misfit was obtained in the proximal zone.
